# Ointment-Based Combination of *Dittrichia viscosa* L. and *Marrubium vulgare* L. Accelerate Burn Wound Healing

**DOI:** 10.3390/ph15030289

**Published:** 2022-02-25

**Authors:** Ibrahim Mssillou, Abdelkrim Agour, Meryem Slighoua, Mohamed Chebaibi, Fatima Ez-Zahra Amrati, Samar Zuhair Alshawwa, Omkulthom Al kamaly, Abdelfattah El Moussaoui, Badiaa Lyoussi, Elhoussine Derwich

**Affiliations:** 1 Laboratory of Natural Substances, Pharmacology, Environment, Modeling, Health and Quality of Life (SNAMOPEQ), Faculty of Sciences Dhar El Mahraz, Sidi Mohamed Ben Abdellah University, Fez 30050, Morocco; abdelkrimagour1@gmail.com (A.A.); lyoussi@gmail.com (B.L.); elhoussinederwich@yahoo.fr (E.D.); 2 Laboratory of Biotechnology, Health, Agrofood and Environment (LBEAS), Faculty of Sciences Dhar El Mahraz, Sidi Mohamed Ben Abdellah University, Fez 30000, Morocco; slighoua.meryem@gmail.com (M.S.); fatima.ezzahra.amrati@gmail.com (F.E.-Z.A.); talhaouisvi@gmail.com (A.E.M.); 3 Biomedical and Translational Research Laboratory, Faculty of Medicine and Pharmacy of the Fez, University of Sidi Mohamed Ben Abdellah, BP 1893, Km 22, Road Sidi Harazem, Fez 30070, Morocco; mohamed.chebaibi@yahoo.fr; 4 Department of Pharmaceutical Sciences, College of Pharmacy, Princess Nourah Bint Abdulrahman University, P.O. Box 84428, Riyadh 11671, Saudi Arabia; szalshawwa@pnu.edu.sa; 5 Unity of GC/MS and GC, City of Innovation, Sidi Mohamed Ben Abdellah University, Fez 30000, Morocco

**Keywords:** *D. viscosa*, *M. vulgare*, analgesic, anti-inflammatory, wound healing

## Abstract

Burns constitute a major challenge in medical science, and plants can be part of the solution. *Dittrichia viscosa* L. (Asteraceae) and *Marrubium vulgare* L. (Lamiaceae) are widely used in Moroccan traditional medicine to treat several diseases and possess high potency to cure wounds. This study aimed to investigate in vivo the analgesic, anti-inflammatory, and burn-healing effects of both plants and their mixture. The hydro-ethanolic extract of both plants was analyzed using high-performance liquid chromatography with diode-array detection (HPLC-DAD). Burns were conducted on dorsal part of rats, and the wound healing process was evaluated during 21 days. Gallic acid, caffeic acid, ferulic acid, and quercetin were identified in *M. vulgare* extract. The analysis recorded the presence of caffeic acid, rosmarinic acid, rutin, and quercetin in *D. viscosa*. The group treated with the mixture showed the lowest abdominal contractions (30.4 ± 7.52) with the highest percentage of inhibition (69.12 ± 7.04%). The inhibition of paw inflammation for *M. vulgare* was 47.65%, followed by *D. viscosa* (33.86%) and the mixture (30.41%). The mixture showed the highest wound contraction at day 7 (33.16 ± 14.33%) and day 14 (87.54 ± 3.98%). *D. viscosa* showed the highest wound contraction on the 21st day (99.28 ± 0.44%). In conclusion, both plants and their combination showed promising results for burn healing.

## 1. Introduction

Wounds are the most frequent disease in the category of skin and subcutaneous ailments, and in addition to the sanitary damage, non-healing wounds can generate massive expenditures ranging from USD 28.1 to USD 96.8 billion in the United States alone [1,2]. Wounds are defined as a damage in the cellular and anatomical structure of vital tissue, mainly the skin and the surface of internal organs. Wounds can be produced by incision, laceration, abrasion, puncture, burns, contusion, and hematoma, as well as crush injuries [3,4,5]. The healing process of wounds includes several phases, such as inflammation, hemostasis re-epithelialization, tissue remodeling, formation of granulation (intervention of keratinocytes, fibroblast migration, and angiogenesis) and proliferation. This process involves the participation of several mesenchymal and epithelial cells [6]. The non-healing wounds can be generated by any disorder in one of these phases [7]. Non-healing wounds are considered as chronic after 4 weeks, and generally the healing process is stopped in the inflammatory phase in this type of wound [8]. Burns can be produced by heat, radioactivity, and electricity, and they are considered to be one of the most severe wounds. This type of injury causes critical damage to the tissue and exposed cells, and it can cause physical and physiological damage and can result in enormous complications if not treated well and at the right time [9,10].

Pain and inflammation are directly related to the wound process; moreover, the most important reasons for which medicines and drugs are used are to reduce the severity of pain and the damage caused by inflammation. Pain and inflammation are generally produced because of wounds, injuries, and several diseases, such as atherosclerosis, rheumatoid, and infectious diseases. Pain and inflammation can also be produced as the result of an auto-immune response [11,12]. The auto-immune response related to pain and inflammation is a normal and positive response that initiates the healing process [13].

Plants with therapeutic effects have been relied on since ancient times to treat many diseases, and plants were recognized as an indispensable source of natural compounds such as triterpenes, flavonoids, phytosterols, and polyphenols [14,15,16]. Medicinal plants and their bioactive compounds, especially phenolic acids and flavonoids, are widely reported to cure wounds and ameliorate the healing process by interfering with cyclooxygenase and prostaglandin biosynthesis [17,18,19,20]. Many studies have proved the analgesic and the anti-inflammatory potential of several medicinal plants [21,22,23,24]. Among these plants, *Dittrichia viscosa* L., a perennial plant (Asteraceae), is characterized by the production of numerous bioactive compounds such as flavonoids, phenolic acids, sesquiterpene lactones, monoterpenes, and triterpenoids [25,26]. *D. viscosa* was reported to possess antioxidant, cytotoxic, antimicrobial, nematicidal, and insecticidal activities. The analgesic, anti-inflammatory and wound healing activities of this plant were proved [25,27]. *Marrubium vulgare* L. (horehound) is an herbaceous medicinal plant native to Europe, northern Africa, and southwestern and central Asia, known for its curative effects and its richness of bioactive compounds i.e., flavonoids, triterpenes, alkaloids, polyphenols, amino acids, polysaccharides, and tannins. *M. vulgare* is widely used in folk medicine to treat several diseases, and it has been reported to possess antioxidant, antibacterial, and antifungal activities; in addition, antidiabetic, anti-inflammatory, analgesic, and wound healing activity was also reported [28,29,30,31,32].

Based on our knowledge, there are no data or studies reporting on the burn-healing, analgesic, or anti-inflammatory activities of these mentioned plants from Morocco or their combination. Therefore, this study aimed to draw attention to the application of these plants as an alternative therapy for wound and burn healing and to explore their analgesic and anti-inflammatory potential, as well as their phytochemical composition using high-performance liquid chromatography with diode-array detection (HPLC-DAD).

## 2. Results

### 2.1. HPLC-DAD Analysis

**Figure 1:**
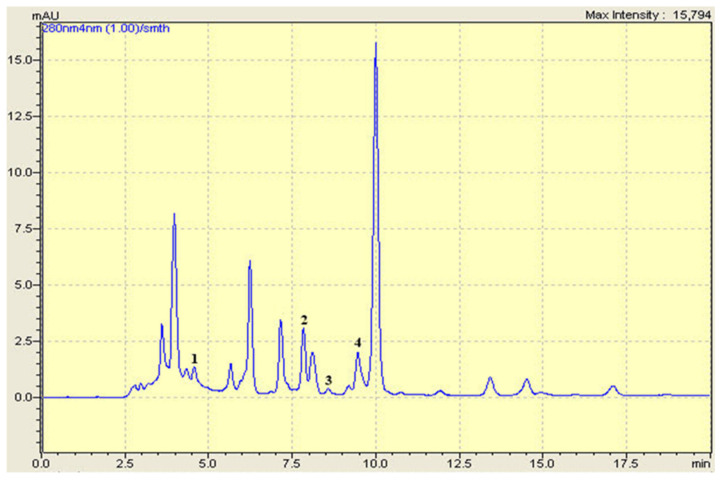
HPLC-DAD chromatogram of *M. vulgare* hydro-ethanolic extract. (1) Gallic acid, (2) Caffeic acid, (3) Ferulic acid, (4) Quercetin.

**Figure 2:**
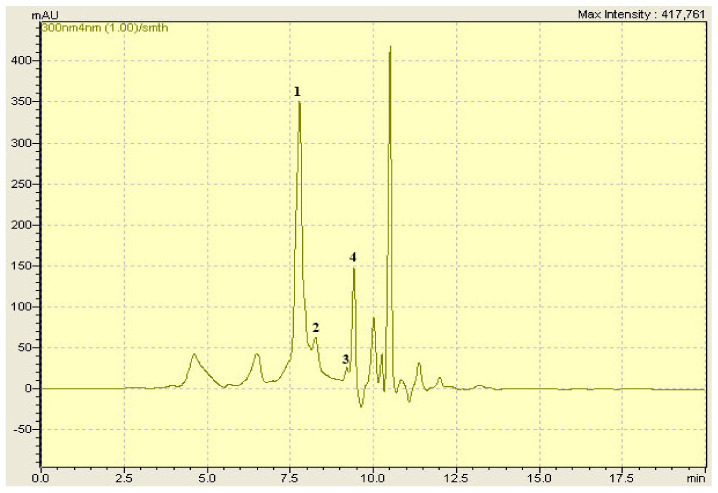
HPLC-DAD chromatogram of *D. viscosa* hydro-ethanolic extract. (1) Caffeic acid, (2) Rosmarinic acid, (3) Rutin, (4) Quercetin.

Both investigated extracts were analyzed using HPLC-DAD. The analysis revealed the presence of four phenolic compounds in each extract (Figure 1 and Figure 2). Gallic acid, caffeic acid, ferulic acid, and quercetin were identified in *M. vulgare*. The analysis showed the presence of caffeic acid, rosmarinic acid, rutin, and quercetin in *D. viscosa*. In general, six different phenolics compounds were identified in both extracts (Table 1). Due to the lack of adequate standards, the compounds contained in the extracts were not all detected.

### 2.2. Analgesic Activity

**Figure 3:**
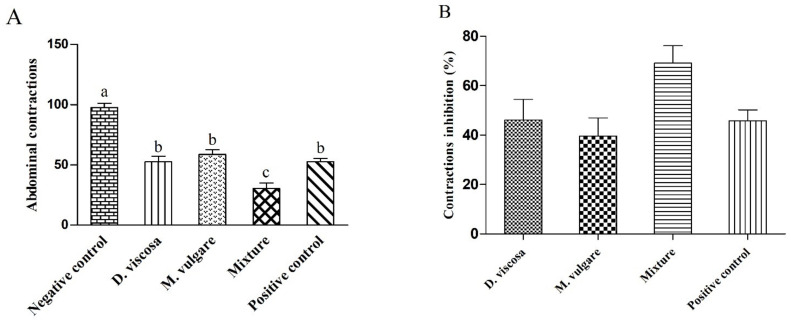
Analgesic effect of hydro-ethanolic extracts from *D. viscosa*, *M. vulgare*, and their mixture compared with the control groups. (**A**) Number of abdominal contractions. (**B**) Inhibition of abdominal contractions in the treated groups. Different letters above bars indicate significant difference at *p* < 0.05 between treated groups (ANOVA one-way followed by Tukey test). The data represent the mean ± SD.

The intraperitoneal injection of acetic acid induced pain on the dorsal part of mice, and the number of abdominal contractions was calculated for 30 min. As summarized in Figure 3, the results confirmed the reduction in pain due to the oral administration of our extracts (500 mg/kg), and the number of abdominal contractions was less compared with the negative control (97.8 ± 6.24). *D. viscosa* (52.6 ± 7.68) showed more effectiveness than *M. vulgare* (58.8 ± 6.64). The mixture (30.4 ± 7.52) presented a higher inhibition of dorsal contractions than both plants and even more than Tramadol^®^ (52.8 ± 4.56). Inhibition of contractions (%) for each extract is presented in Figure 2. The mixture had the highest activity (69.12 ± 7.04%), followed by *D. viscosa* (46.09 ± 8.43%) and Tramadol^®^ (45.86 ± 4.38%), and finally *M. vulgare*, which had the lowest analgesic effect (39.62 ± 7.31%).

### 2.3. Carrageenan-Induced Rat Paw Test

**Figure 4:**
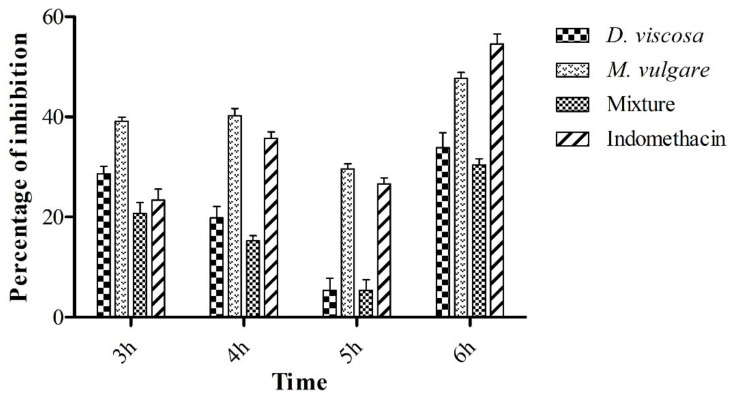
Effect of different hydro-ethanolic extracts administered orally on carrageenan-induced edema in rats. The percentage of inhibition concerning the circumference in the right paw of rats. The data represent the mean ± SD.

The anti-inflammatory effect of *D. viscosa*, *M. vulgare,* and their mixture (500 mg/Kg) was evaluated using the carrageenan-induced rat paw test. The circumference/diameter of rat’s paw (*n* = 5) and the percentage of the paw volume (cm) at 3, 4, 5, and 6 h after carrageenan (1%) injection are respectively demonstrated in Table 2 and Figure 4. We observe from the table the increase in paw volume as a function of time, whereas the volume of paw reached the maximum at the fourth hour for all extracts. After 4 h from the carrageenan injection, the vehicle (NaCl (0.9%)), presented the highest circumference (3.04 ± 0.11 cm). Regarding the groups treated with *D. viscosa* (2.82 ± 0.10 cm), *M. vulgare* (2.68 ± 0.10 cm), the mixture (2.66 ± 0.15 cm), and Indomethacin^®^ (2.74 ± 0.04 cm), it can be ascertained that the extracts possess an anti-inflammatory effect. Furthermore, the group treated with the mixture showed the highest inhibition of edema at the time of the maximum inflammation (4 h). After 6 h, all the extracts showed a significant reduction in the size of the paw at *p* < 0.05 compared with the control.

Moreover, Figure 4, shows that after 6 h passed from the carrageenan injection, Indomethacin^®^ (10 mg/mL) presented the highest inhibition of edema (54.55%), followed by *M. vulgare* (47.65%) and *D. viscosa* (33.86%), and the mixture produced only 30.41% of edema inhibition.

### 2.4. Wound Healing Activity

**Figure 5:**
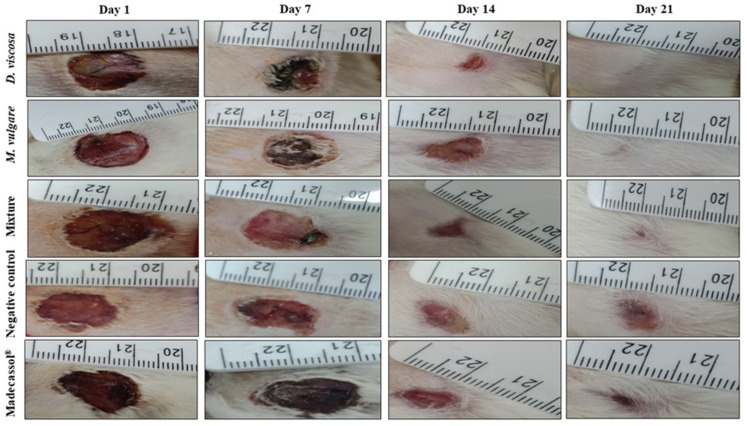
Aspect and photographical representation of burn healing process and morphological appearance of the wounds in groups treated with *M. vulgare*, *D. viscosa*, the mixture, and the control group from day 1 until day 21.

As shown in Table 3, topical application of ointments from *D. viscosa*, *M. vulgare,* and their mixture showed significant progressive wound healing compared with the controls. Figure 5 shows the photographic progress of wound healing of ointments and the vehicle groups. A significant wound closure was recorded for all the ointments from the 1st day until the 21st day. Topical application of the plants and the mixture led to wound closure at day 21; however, in the untreated group (only Vaseline^®^) and also the group treated with Madecassol^®^, wounds did not close completely on the last day of treatment.

**Figure 6:**
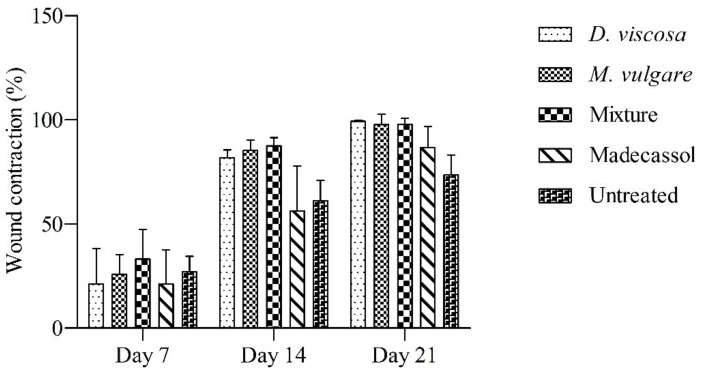
Burn healing effect of *D. viscosa*, *M. vulgare*, the mixture applied dermally, the untreated group, and the group treated with Madecassol^®^. The size of wounds on the first day of the experiment was used as reference. The data represent the mean ± SD.

Results of wound contractions during the 7th, 14th, and 21st day are presented in Figure 6. The group treated with the mixture showed the highest wound contractions on the 7th (33.16 ± 14.33%) and 14th day (87.54 ± 3.98%). On day 21, *D. viscosa* showed the highest wound contractions (99.28 ± 0.44%), followed by *M. vulgare* (97.78 ± 4.95%) and the mixture (97.96 ± 2.91%). A wound contraction of 86.74 ± 9.9% was recorded in the group treated with Madecassol^®^ on the 21st day. The lowest wound contractions were observed in the vehicle group (73.59 ± 9.59%), where the wounds did not completely close.

## 3. Discussion

Several studies reported that phenolic compounds possess many biological activities, such as antioxidant, antimicrobial, analgesic, anti-inflammatory, and wound healing properties [33,34]. Gallic acid, caffeic acid, ferulic acid, quercetin, rosmarinic acid, and rutin identified in *D. viscosa* and *M. vulagre* have been reported in other studies as the main phenolic compounds of these plants [28,35].

In the study of Ouahchia et al. [36], *D. viscosa* was reported to possess analgesic activity with 93.39% of writhing inhibition at a dose of 800 mg/kg. Additionally, in another study conducted by Martin et al. [37], *D. viscosa* proved its analgesic activity. Concerning *M. vulgare,* many studies reported on its ability to reduce pain. The study performed by de Souza et al. [38] showed significant analgesic activity using the hydroalcoholic extract from *M. vulgare* in both acetic acid and formalin protocols. In the same context, Marrubiin derivatives extracted from *M. vulgare* were examined for their analgesic potential in several models, and results showed an inhibition ranging from 48 ± 1% to 92 ± 1% [39]. Therefore, the high inhibitory effect produced by the mixture could be due to the synergistic interactions of the plants used. In the literature, several studies support the advantage of using plants in combination rather than individual herbal extracts, due to the high efficacy and the negligible side effects and toxicity [40,41].

Phenolic acids (gallic acid, caffeic acid, rosmarinic acid, and ferulic acid) and flavonoids (rutin, quercetin) identified in both plants were all reported to possess analgesic activity in several models [42,43,44]. Prostaglandin, serotonin, and bradykinin are the principal endogenous mediators that trigger pain by the elevation of the stimuli of peripheral nerves resulting in the activation of nociceptors [45]. It was proven that the secretion of prostaglandins is regulated by arachidonic acid. The interleukin-1β (IL-1β) and cyclooxygenase enzymes (COX-1 and COX-2) are the main intermediates in the production process of prostaglandin from arachidonic acid [46]. Some important flavonoids such as rutin, quercetin, myricetin, kaempferol, fisetin, and flavones (luteolin, apigenin) were described as being inhibitors of the cyclooxygenase and/or the 5-lipoxygenase pathways of arachidonate metabolism [47,48]. The analgesic activity observed in both extracts and their mixture could be due to the presence of phenolic acids and flavonoids in their chemical composition.

The carrageenan-induced paw edema protocol is the most suitable model using in vivo animals to investigate the anti-inflammatory effect of natural products [49]. The metabolism of arachidonic acid causes an increase in tissue fluid and extravasation of plasma and neutrophils, which in turn leads to inflammation [50]. In early hyperemia, the carrageenan injection results in the secretion of mediators such as bradykinin, histamine, and serotonin, and after 2–3 h of injection, the paw circumstance reaches the highest level, and after that, it starts falling [51]. The second phase is characterized by the release of prostaglandin and migration of leukocytes into the inflammation site [52].

*D. viscosa* was recorded to possess an anti-inflammatory effect, and its inhibitory effect on lipid mediators such as phospholidase A2 and cyclooxygenase was confirmed [53]. In this regard, the anti-inflammatory activity of *M. vulgare* and its catalyzation effects on cyclooxygenase and prostaglandin biosynthesis was also proved [54]. It is necessary to mention that phenolic acids and flavonoids are principally involved in the anti-inflammatory process of both plants [47]. The hydroxyl group of flavonoids was reported to affect oral anti-inflammatory activity [55]. Baicalein is another flavonoid that was reported to possess anti-inflammatory properties and inhibition of leukotriene C4 biosynthesis [56].

Burn healing is a complex mechanism that requires the stimulation of several cell types as intermediaries to result in dermic and epidermic tissue repair. Mainly, the inflammatory response requires the intervention of neutrophils and monocytes, and this phase is directly linked to the process of wound healing [57]. In the granulation tissue, the mesenchymal cells are very important in tissue remodeling. In addition to the proliferation and migration of epithelial cells, fibroblasts proliferation, synthesis of collagen, and the action of keratinocytes are mainly involved in the healing process [58].

In modern medicine, several drugs (1–3%) are prescribed for the treatment of wounds, and almost over 30% of formulations in traditional medicine are linked to skin disorders, which reveals the high effectiveness of medicinal plants to promote the discovery of new drugs with wound healing properties [59]. Despite the potency of individual medicinal plants in wound care, the polyherbal formulations are more encouraged due to their high effectiveness and low number of side effects [60,61].

In the present study, *D. viscosa* and *M. vulgare* and their mixture were examined for their wound healing activity. Observing the results, it appears that these plants certainly accelerate the healing process compared with the control groups. Looking at the literature it is obviously clear that these plants stimulate the healing mechanism and that they possess a high potency to cure wounds [62,63]. In the same context, it has been proven that flavonoids play an important role in the wound healing process. Furthermore, phenolic acids, such as caffeic acid, promote fibroblast levels, leading to remolding injured tissue [17,20]. It has been proven that quercetin performs a crucial action on the collagen matrices for dermal wound healing processes [64], and the effectiveness of ferulic acid in the healing process was also proved [65]. Other flavonoids, such us myricetin, naringenin, isoliquiritin, curcumin, and apigenin, were also reported to possess wound healing activity [66,67].

## 4. Materials and Methods

### 4.1. Harvesting and Identification of Plants

The plants used in this study were collected around the city of Fez in Morocco (34°03′41.3″ N 5°03′45.5″ W) in December 2020. *D. viscosa* L. and *M. vulagare* L. were brought to the laboratory and identification was carried out by Pr. Bari Amina, a professor at Sidi Mohamed Ben Abdellah university. A voucher sample was specified for each plant, *D. viscosa* L (DV20201214) and *M. vulagare* L (RM001617).

### 4.2. Preparation of Hydro-Ethanolic Extracts and the Mixture

The leaves were cut from the fresh plants and left for a week in the laboratory to dry; then the leaves were grinded to fine particles using a Waring^®^ blender. To prepare the hydro-ethanolic extracts, 30 g of each plant was macerated using 210 mL of pure ethanol (99%) and 90 mL of distilled water at a rate of 70% (*v*/*v*) and 30% (*v*/*v*), respectively. The maceration was performed for 72 h at laboratory ambient temperature. The macerates were filtered using a Whatman paper no◦1 and evaporated with a rotary evaporator at 37 °C. The mixture was prepared by mixing the hydro-ethanolic extract of *D. viscosa* and *M. vulgare* at a rate of 50% (*w*/*w*).

### 4.3. Animal Handling and Housing

Male Wistar rats used in this study were obtained from the ECWP (Emirates Wildlife Propagation Center) in Missour-Morocco, with a weight ranging between 120 g and 140 g. Male mice used for the analgesic activity were obtained from the animal house in the Department of Biology, Faculty of Sciences Dhar El-Mahraz, Sidi Mohamed Ben Abdellah University, Fez, Morocco. Mice were characterized by a weight between 35 g and 45 g. The animals housing was assisted in the animal house in cages, where the temperature was fixed at 28–32 °C and 50% to 55% for relative humidity. Rats and mice had free access to food and water and were subjected to a photoperiod day/night cycle (~12/12 h). The study procedure and activities carried out in this research were performed according to the guidelines of the internationally accepted standard of the European community for the protection of animals used for experimental purposes (council directive of 24 November 1986) [68], and the standard of animal housing and handling of the faculty of sciences of Fez, Morocco, committee (04/2021/SNAMOPEQ).

### 4.4. HPLC-DAD Analysis

Gallic acid, vanillin, rutin, caffeic acid, rosmarinic acid, furilic acid, luteolin, catechic, arbutin, and quercetin were purchased from Sigma-Aldrich (Germany). An amount of 100 ppm for each standard was prepared in methanol.

Reverse phase high-performance liquid chromatography (HPLC) was used to analyze the hydro-ethanolic extract of *D. viscosa* and *M. vulgare*. A shimadzu HPLC system was used with an analytical column (C18) SGE 250 × 4.6 mm SS Exsil ODS 5 μm. The separation was carried out in gradient mode, using two solvents; A (water/acetic acid) (97.5: 2.5, *v*/*v*) and B (methanol/acetonitrile) (50: 50, *v*/*v*) according to the following elution gradient: (5% A, 95% B) at 0 min, (30% A, 70% B) at 6.25 min, (35% A, 65% B) at 12.5 min, (70% A, 30% B) at 16.25 min, (100% A, 0% B) at 17.5 min, and (5% A, 95% B) at 18.75 min.

Flow rate was 1 mL.min^−1^, and the injection volume was equal to 20 µL. The detection was performed between 200 and 800 nm.

### 4.5. Analgesic Activity

The analgesic activity was evaluated using the acetic acid method described by Karbab et al. [69]. In this test, male mice were divided into 5 groups with 5 mice in each group. In group 1 (negative control), the mice were treated orally with NaCl (0.9%). Groups 2–4 were treated, respectively, with the hydroethanolic extract (500 mg/kg) of *D. viscosa*, *M. vulgare,* and the mixture. Group 5 (positive control) was treated with Tramadol^®^ (50 mg). After 90 min of oral administration dedicated to each group, all the mice received via intraperitoneal injection a dose of 10 mg/mL of acetic acid at 0.7% (*v*/*v*). Counting of abdominal contractions was performed after 5 min of the injection of acetic acid during 30 min. The formula below was used to calculate the percentage of inhibition of abdominal contractions:

PI% = (M_n_ − M_t_)/M_n_ × 100

M_n_: mean number of abdominal contractions in the negative control group, M_t_: mean number of abdominal contractions in each group treated with the extracts or Tramadol.

### 4.6. Carrageenan-Induced Rat Paw Inflammation

To investigate the anti-inflammatory activity of *D. viscosa*, *M. vulgare,* and their mixture, we followed the protocol described in the study conducted by Winter et al. [70]. Wistar rats were divided into 5 groups (5 rats in each group). Group 1 corresponded to the negative control and received only NaCl at 0.9%. The second to the fourth groups received orally the hydro-ethanolic extract of *D. viscosa*, *M. vulgare*, and their mixture, respectively, at a dose of 500 mg/Kg. The fifth group used as positive control, received Indomethacin^®^ (10 mg/kg). The circumference of the right paw of all rats was measured before the injection of carrageenan (1%); then the measurement was performed after 3, 4, 5, and 6 h of the injection. The percentage inhibition of inflammation was calculated according to the equation below:

PI%= [(*C*_t_−*C*_0_) Control − (*C*_t_ − *C*_0_) treated/(*C*_t_ − *C*_0_) Control] × 100

*C*_0_: The mean paw circumference before injection.

*C*_t_: The mean paw circumference after carrageenan injection at a given time.

### 4.7. Wound Healing Test

#### 4.7.1. Ointments Preparation

Ointment of each plant and the mixture was prepared at 10% (*w*/*w*) by melting 1 g of the hydro-ethanolic extract in 9 g of Vaseline^®^. The quantity of the hydro-ethanolic extract was added carefully to Vaseline^®^ in a beaker at 50 °C using a water bath, with continuous stirring until homogenous. The ointment of the mixture was formed with 500 mg of each plant extract at the same rate and following the same procedure. Ointments were stored in airtight containers at 4 °C.

#### 4.7.2. Burn Wound Induction

Burn induction on dorsal part was performed according to the protocol described by Heidari et al. [71]. A total of 25 rats were dedicated for this test and were divided into 5 groups, 5 rats for each group. The first group received Vaseline^®^ as a negative control; the second to the fourth group received the ointment of *D. viscosa*, *M. vulgare,* and the mixture, respectively. The fifth group was treated with a healing ointment (Madecassol^®^ at (1%)) as a positive control. The dorsal part of all the rats was shaved with an electric clipper. The anesthesia was performed intraperitoneally using pentobarbital (50 mg/kg). The induction of burns was carried out on the shaved part of rats using an aluminum rod (1.7 cm) that was heated to 110 °C and placed on the burn induction zone with a pressure of 1 atm for 10 s. Treatment was started after 24 h of burn induction. The treatment was applied daily, covering the entire burned area with the ointments for 21 days. The burned area was photographed for all rats using a digital camera and a ruler as a scale. The contraction rate of wounds was measured by the analysis of images of burned area using ImageJ software and the following formula:

WC (%) = [(WS0 − WSSD)/WS0] × 100

WC (%): Percentage of wound contraction

WS_0_: Wound size in the first day

WS_SD_: Wound size in each specific day

### 4.8. Statistical Analysis

Results were analyzed with one-way ANOVA test (GraphPad Prism software version 5), and *p* < 0.05 showed statistical significance. Results are expressed as mean ± standard deviation.

## 5. Conclusions

The present study evaluated the burn healing activity of *D. viscosa*, *M. vulgare,* and their mixture. All the extracts presented high effectiveness to cure burns and promoted the healing process. Furthermore, as pain and inflammation are linked to the healing process, the analgesic and the anti-inflammatory effects were also examined, and the studied plants showed promising results to reduce pain and inflammation. The presence of phenolic acids such as ferulic acid and caffeic acid, and flavonoids—namely, quercetin and rutin—increased the healing process of plants, as reported and proved in other studies. The results proved that the combination of both plants supports the use of polyherbal formulations to treat such diseases with greater efficiency and the avoidance of sides effects.

## Figures and Tables

**Table 1:** Phenolic compounds identified in *D. viscosa* and *M. vulgare* with their retention time (RT).

Phenolic Compounds	Formula	RT (min)
Gallic acid	C_7_H_6_O_5_	4.69
Caffeic acid	C_9_H_8_O_4_	7.777
Rosmarinic acid	C_18_H_16_O_8_	8.300
Ferulic acid	C_10_H_10_O_4_	8.40
Rutin	C_27_H_30_O_16_	9.027
Quercetin	C_15_H_10_O_7_	9.403

**Table 2:** Rats paw diameter (cm) of treated groups and the control (NaCl 0.9%) before the injection of carrageenan and at 3 h, 4 h, 5 h, and 6 h after the injection.

	Diameter in cm				
Treatment	0 h	3 h	4 h	5 h	6 h
NaCl 0.9%	2.16 ± 0.04	2.92 ± 0.07 ^a^	3.04 ± 0.11 ^a^	2.82 ± 0.06 ^a^	2.74 ± 0.04 ^a^
*D. viscosa*	2.22 ± 0.14	2.58 ± 0.10 ^b^	2.82 ± 0.10 ^a,b^	2.62 ± 0.14 ^a,b^	2.36 ± 0.07 ^b^
*M. vulgare*	2.26 ± 0.12	2.54 ± 0.08 ^b^	2.68 ± 0.10 ^b^	2.50 ± 0.12 ^b^	2.32 ± 0.10 ^b^
Mixture	2.02 ± 0.03	2.44 ± 0.08 ^b,c^	2.66 ± 0.15 ^b^	2.56 ± 0.08 ^b^	2.18 ± 0.09 ^b^
Indomethacin	2.28 ± 0.05	2.68 ± 0.05 ^b,d^	2.74 ± 0.04 ^b^	2.54 ± 0.07 ^b^	2.30 ± 0.06 ^b^

Values in the same column from the third hour with a different letter above indicate a significant difference at *p* < 0.05 in one-way ANOVA analysis, followed by Tukey’s test. The data represent the mean ± SD.

**Table 3:** Wound size in cm^2^ of each group of rats from day 1 till day 21.

	Wound Size in cm^2^			
Treatments	Day 1	Day 7	Day 14	Day 21
*D. viscosa* (10%)	1.73 ± 0.16 ^a^	1.43 ± 0.4 ^a^	0.31 ± 0.08 ^b^	0.01 ± 0.005 ^c^
*M. vulgare* (10%)	2.58 ± 0.48 ^a^	1.91 ± 0.48 ^a^	0.39 ± 0.2 ^b^	0.07 ± 0.16 ^c^
Mixture (10%)	2.31 ± 0.4 ^a^	1.51 ± 0.25 ^a^	0.3 ± 0.16 ^b^	0.05 ± 0.09 ^c^
Madecassol (1%)	1.45 ± 0.55 ^a^	1.08 ± 0.19 ^a^	0.57 ± 0.17 ^b^	0.15 ± 0.1 ^c^
Negative control	1.85 ± 0.6 ^a^	1.34 ± 0.43 ^a^	0.68 ± 0.12 ^b^	0.45 ± 0.08 ^b^

Values in the same row with a different letter above indicate a significant difference at *p* < 0.05 in one-way ANOVA analysis, followed by Tukey’s test. The data represent the mean ± SD.

## Data Availability

Data is contained within the article.
